# A New Use for Petroleum

**Published:** 1865-02

**Authors:** 


A New Use for Petroleum.—Dr. Decaisne, of Antwerp, announces that the itch may be cured instantaneously by simply applying (without rubbing) petroleum to the parts affected. The mere emanations of that oil are sufficient to disinfect the patient's clothes, and Dr. Decaisne adds that all other parasites of the human body may be destroyed immediately by the same means.

